# Financial Incentive Does Not Affect P300 in the Complex Trial Protocol (CTP) Version of the Concealed Information Test (CIT) in Malingering Detection. II. Uninstructed Subjects

**DOI:** 10.3389/fpsyt.2019.00189

**Published:** 2019-04-15

**Authors:** J. Peter Rosenfeld, Elena Davydova, Elena Labkovsky, Anne Ward

**Affiliations:** Department of Psychology, Northwestern University, Evanston, IL, United States

**Keywords:** P300 CIT, deception, motivation, incentive, complex trial protocol

## Abstract

Well-known research showed that the skin conductance response (SCR) of the Autonomic Nervous System (ANS) in the Concealed Information Test (CIT) is usually augmented in participants who are financially and motivationally incentivized to beat the CIT. This is not what happens with Reaction Time (RT)-based CITs, P300 CITs based on the 3-stimulus protocol, nor on the P300-based complex trial protocol for detection of malingering (however these tests differ from forensic CITs). The present report follows up the Rosenfeld et al. (1, 2) study of motivated malingerers *instructed* how to beat the test, with *uninstructed* motivated (paid and unpaid) and unmotivated (“simple malingering”) subjects, using episodic and semantic memory probes. The Test of Memory Malingering (TOMM) validated behavioral differences among groups. The “CIT effect” (probe-minus-irrelevant P300 differences) did *not* differ among incentive groups, although as previously, semantic memory-evoked P300s exceeded episodic memory evoked P300s. An effect of specific test-beating instructions was found to enhance the CIT effect for semantic information.

## Introduction

The Concealed Information Test [CIT, (3), previously known as the Guilty Knowledge Test or GKT] has been studied for half a century; [for reviews, see (4–6)]. In this test, there are at least two kinds of stimuli randomly presented regarding order to participants: The (1) *probes* are the items expected to be remembered; they are often from a crime scene in a forensic scenario—such as, a stolen diamond necklace. The (2) *Irrelevant* stimuli are other comparably valuable items (a watch, a bracelet, a broach, etc.) which are from the same category as the probe (jewelry), but are not identical to it, so are unrecognized by the thief as the stolen item. The probe *is* recognized, and therefore elicits a larger physiological response in only the knowledgeable participant. To innocent suspects, the probe is just another irrelevant so elicits a smaller or no physiological response.

The traditional responses examined in the CIT are autonomic nervous system (ANS) responses such as, Skin Conductance Response (SCR), respiration pattern, and cardiac responses. More recently, the P300 component of the event-related potential (ERP) and fMRI have been utilized [see (6–8). When P300 is used, the probes are presented infrequently, e.g., probability = *p* = 0.15, and the irrelevants are presented frequently, e.g., *p* = 0.7, and a third stimulus type—the target (*p* = 0.15)—that has a unique response requirement—is also used, mainly to assure attention].

In a recent meta-analysis, Meijer et al. (5) noted that many workers have reported that motivation and incentive typically increase the CIT effect in the SCR measure of the ANS. However, this does not happen with reaction time (RT) measures of CIT effects (9–11).

With respect to P300-based CITs, Meijer et al. (5) stated that “The bulk of CIT studies based on P300 did not use motivational instructions.” We agree with this, since most of those studies were from this lab where we never reported effects of motivation on P300 in several reports. (That is, P300 amplitudes in CIT studies with incentivized subjects appear to be in the same range as they are in those studies without financial incentive). This was formally confirmed in Ellwanger et al. (12): Participants in a truth-telling group, instructed to do their best on P300 tests (involving semantic, as well as incidentally acquired, episodic memory), were compared to a motivated/incentivized “dishonest” group offered a $10 reward to “beat the test.” There were no significant P300 differences found: The sensitivity of the truth tellers was 0.74, vs. 0.73 for the incentivized dishonest group. This is clear evidence that the motivational manipulation of offering a $10 reward for beating the test did not affect the CIT effect or sensitivity of the P300-based CIT. This study utilized the older “3-stimulus protocol” [3SP, (7)]. We want to emphasize, however, that the malingering protocol that detects feigned cognitive deficit about autobiographical knowledge has critical differences from the forensic CIT protocol that detects feigned ignorance of crime details, and this fact makes it difficult to generalize from malingering data to forensic CIT data. We will re-visit this issue in the discussion.

It is noted that the present and previous tests of malingering use both verbal/behavioral tests as well as P300 data, typically with a comparative aim. The verbal/behavioral tests are designed to entrap malingerers by giving them an explicit test of autobiographical memory recognition, which is easy, but appears to be more difficult, and on which they typically, but not reliably, score poorly. Because of dissatisfaction with these tests among neuropsychologists, physiological measures, especially P300, were introduced to detect malingered cognitive deficit in closed head injury (CHI) patients; (13–16). P300s are reliably evoked in response to recognized information, which has prompted their use in forensic situations, (7). It followed that P300 tests might be profitably used in detecting malingering: Malingerers may state that they forgot a learned word but if the word elicits a P300, this strongly suggests that the denied word is recognized despite the behavioral denial.

Recently, Rosenfeld et al. (1) formally observed a similar result—no effects of financial incentive manipulations on P300—using the newer, and countermeasure-resistant Complex Trial Protocol (CTP detailed below) for detection of concealed information (17). In this 2017 study (1), there were two groups. Both were motivated to beat the test and instructed specifically how to beat the test, but one group was paid for success and the other was not. Our main finding was that although there were clear, behavioral differences in the malingering *behavior* (on the Test of Memory Malingering, described below, p. 7) of the two groups, these significant effects were not reflected in the ERP data: The “Concealed Information Test (CIT) Effect”—the difference between rare critical probe and frequent irrelevant P300 amplitude– did not differ between groups. Detailed description, comparison and review of the 3SP vs. the CTP is in Rosenfeld (7). Thus, when two groups are motivated to defeat the test and instructed how best to beat it, there is no incremental effect of financial incentive on the P300 CIT effect. Indeed, it may have been the case that since both groups were motivated to beat the test and shown how to beat it, they may have been at a ceiling level of motivation.

Therefore, in the present study, we focus solely on uninstructed participants (Ps), and compare an unpaid, unmotivated “simple malingering” (SM) group to two other groups, both motivated to beat the test, with one paid to do so, and the other, unpaid. We will also compare the paid vs. unpaid, but both motivated, groups. None of the aforementioned studies examined the incremental effect of instructions specifically directed to defeating the tests by simulating malingering. This will be done here by comparing instructed groups of Rosenfeld et al. (1) with two uninstructed groups run here 1 year later on a different participant set by different experimenters.

In both Rosenfeld et al. (1) and Ellwanger et al. (12), the experimental scenario involved the simulated malingering of cognitive (memory) deficits which accompany closed head injury (CHI). As Ellwanger et al. (12) have noted, the simulating normals are not instructed to suppress *all* responses to critical/probe items, which, in contrast, *is* the case with a classical CIT scenario, making scientific comparison (of malingering and forensic scenarios) problematic. Rather, the CHI malingerer is told to imitate the performance of a real CHI patient by not making errors on *all* critical/probe items, but to only about half of them.

In the present paper as well in Rosenfeld et al. (1), we use the Test of Memory Malingering [TOMM, (18)] which is universally regarded today as the gold standard for such tests [(19, 20). See methods for more detail]. This is a familiar study-test protocol where old stimuli are first learned, after which a recognition test for learned (old) vs. new stimuli is given. For a given test item (old or new) a subject can respond either correctly/honestly, or—in a malingering fashion—dishonestly or truly incorrectly. Based on our earlier studies cited above, we expect that paid malingerers will pay closer attention to test items than unpaid subjects will, and so (a), will give more correct than incorrect responses on the TOMM, yet based on Ellwanger et al. (12) and Rosenfeld et al. (1), (b) they will *not* show an effect of financial and other incentivization on the P300 CTP test.

**Figure 1:**
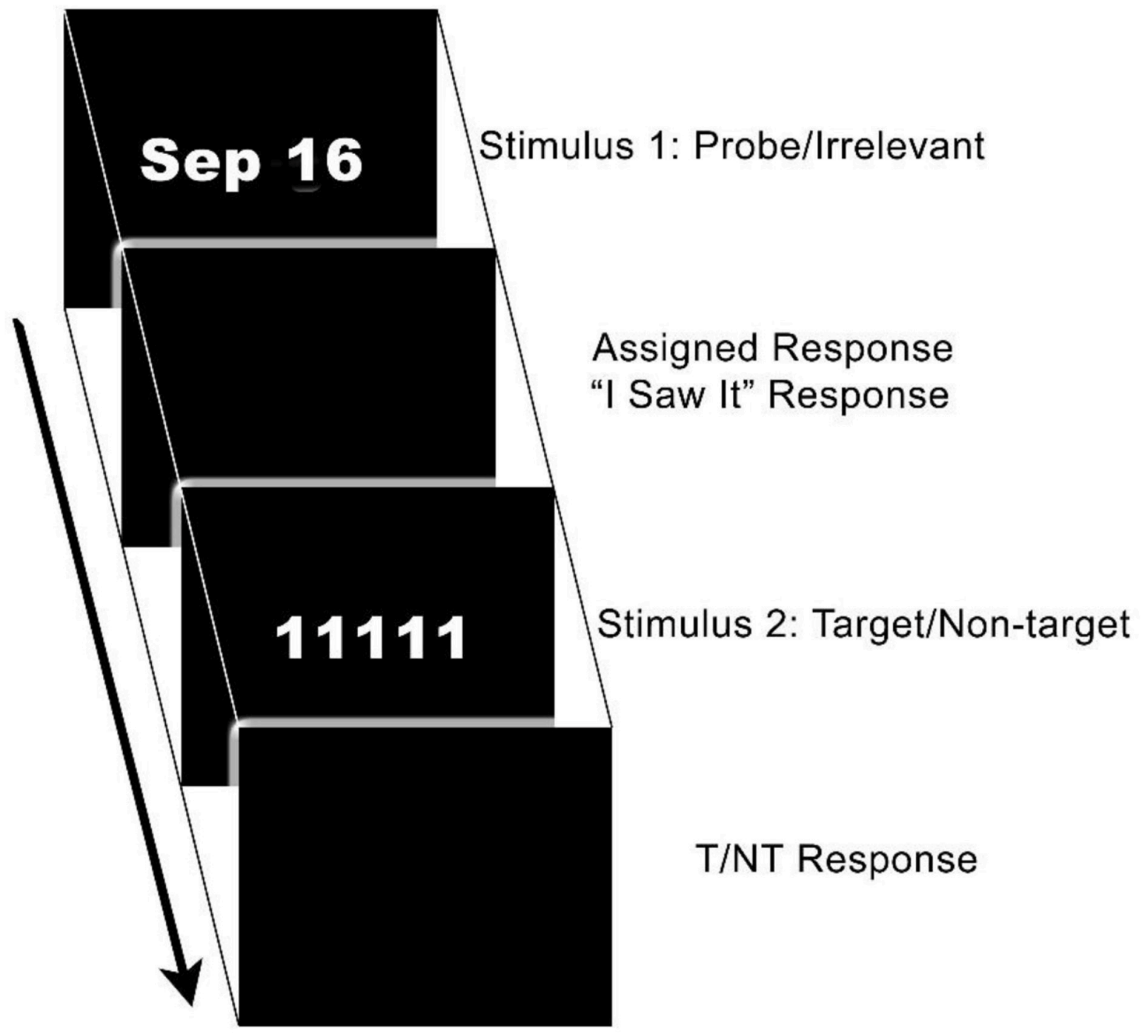
The Complex Trial Protocol event sequence, with a date stimulus as stimulus 1 (probe or irrelevant), then the perception acknowledgment response (“I saw it”), then the target or non-target as stimulus 2, then the target or non-target response. All stimuli are presented for 300 ms each. There is a randomly varying interval of 1300–1800 ms between S1 and S2. There is a 2 s interval between the T/NT response and the next S1 for the next trial.

The background and essence of the CTP is described here: The CTP was designed to address the weaknesses of the original “3-stimulus protocol” (3SP, 17). Rosenfeld et al. suggested that the 3SP generated smaller than usual P300 responses to probes because Ps also make an explicit target decision (i.e., target vs. non-target) on every trial. Although probes do produce a P300 in guilty individuals in the 3SP, the extra job of determining if each presented item is a target weakens attention to probes, and since decision-making absorbs processing resources, it reduces the P300 response to the probe (21, 22). The CTP addresses this issue by separating probe vs. irrelevant and target vs. non-target decisions by ~1 s. In this two-part trial, a simple “I saw it” response is required for the first stimulus (probe or irrelevant), which is followed by a target vs. non-target decision; (see Figure 1, showing a date stimulus 1 [S1] and a subsequent target [“1111”]). The initial stimulus (i.e., probe or irrelevant) requires a unitary “I saw it” button response with the left hand, but the subsequent target-non-target response depends on the second stimulus type (S2), so that differing right-hand mouse buttons correspond with the target (“yes” button) and non-targets (“no” button). Also, targets and non-targets are typically from a different category than probe/irrelevants. Separating the implicit (probe vs. irrelevant) and explicit (target vs. non-target) decisions—*combined* in the 3SP—frees processing resources, resulting in larger P300 responses, and greater differences between probe and irrelevant P300s, thereby improving CM resistance (17). Comparisons of the CTP and the 3SP are detailed in Rosenfeld (7).

## Methods

### Participants

The subjects were recruited from the Northwestern University Introductory Psychology Pool. Participants were mostly college freshmen and sophomores, plus a few juniors and seniors, aged 17–22. The study was consistent with ethical guidelines as it was approved by the Northwestern IRB. There were initially three groups, 2 of 21 each, and one of 22 participants. The groups were formed by random assignment to groups which is expected to assure gender and age balance across groups. The three groups had 14, 16, and 15 females. The group numbers were based on a power analysis directed at having an 80% likelihood of discovering a medium size effect with alpha = 0.05. For all 64 subjects, the mean age = 18.8, SD = 1.4. There was (1) A group told to simulate malingering (SM group) but not to try to beat the test, nor rewarded for same. (2) Two groups told to simulate malingering and encouraged to try to beat the test. One of these groups was unpaid (BtNo) and the other was paid (Bt $) to beat the test. None were instructed how to beat the test as the subjects were in Rosenfeld et al. (1). These subjects were told to duplicate performance of head injury patients by not getting every item wrong, but by answering incorrectly only about half the time. The Supplementary Materials give detailed instructions.

### Procedures

The probe stimuli used in the CITs were the P's birthday (semantic memory) in one block, and the experimenter's name (episodic memory) in a second CIT block; block order was counterbalanced across Ps in both paid and unpaid groups. We do not mean to imply that birthday and experimenter name are the perfect exemplars of the 2 respective memory categories. Other exemplars may have different results. Here, so as to replicate Ellwanger et al. (12) and Rosenfeld et al. (1), exposure to the experimenter's name was as follows: The P was first contacted via an e-mail (sent to arrange the experimental session time) in which the experimenter's name appeared twice. When a P entered the lab, (s)he was greeted at the door with the sentence, “Hi come on in. My name is Elena. I e-mailed you about our appointment.” (The entire verbatim interaction and instructions used with all Ps are seen in the Supplementary Materials). Instructions were given, the subject was asked to look at a list of intended irrelevant date stimuli to be used, and to circle any that were unintentionally and by chance, relevant personally—such as, the birth date of a close acquaintance. This was replaced in the list of irrelevants to be used in the date CIT. After full instructions (in Supplementary Materials) were given, and just before the name block in the P300-CIT, the experimenter asked the P if (s)he remembered the experimenter's name. If P did, the CIT was given. If not, the experimenter repeated her first name while holding up a card with this name. The subject then repeated the name. The P300-CIT followed, and after it, the P was then tested again on the name. All Ps responded correctly. All Ps were also asked after the birthday block if they saw the birthday; all reported that they did.

The next procedure was administration of a modified version of the Test of Memory Malingering [TOMM, (18)], a validated (23, 24) instrument strongly supported by Teichner and Wagner (25) to detect malingering. (They stated: “Results suggest that the TOMM is a useful index for detecting the malingering of memory deficits.”) The TOMM is universally regarded today as the gold standard for tests of memory malingering (19, 20).

The version we used was an abbreviated version of the TOMM as suggested by Hilsabeck et al. (26). The abbreviated TOMM was used in order to assess the malingering manipulation and compare its effects among groups.

The TOMM we used involves a study-test manipulation, with 50 initial exposures of line drawings of common objects in a study block, one by one, followed after about 2 min with a test on 100 more pictures containing the randomly ordered 50 initially studied (“old”) pictures randomly shuffled with 50 novel (“new”) pictures. Ps were instructed to press one button if they recognized the picture, and another if they did not. Thus, there were two types of outcomes (correct and incorrect/faked) on all test trials with *Old* stimuli, and likewise for test trials with *New* stimuli. Ps were still under the malingering instruction set, and were so reminded in the TOMM. (See Supplementary Material).

We note that in the usual clinical version of the TOMM (18), the *test* stimuli are presented as 50 pairs, each containing an old drawing plus a new drawing. This is similar to our test, which is no more difficult than the clinical TOMM, so the norms (26) for the clinical version, probably apply here. They are that a score of 82% or more is probably from a non-malingerer, whereas, a score of 62% or less is from a malingerer.

At this point, all motivated (told to beat the test) Ps were shown their averaged probe and irrelevant P300s, so as to determine with our bootstrap software (described below) whether they were detected in their malingering or not, based on the P300 values. We illustrate the superimposed probe and irrelevant ERPs of guilty vs. Innocent participants in previous studies, and describe how large differences indicate guilt. Moreover, we tell them that the software will output the expected numbers of times in 100 samples that the probe > irrelevant P300. We also tell them that a 90 is required for a guilty diagnosis. Successful members of the paid group were paid. Then all Ps were discharged.

It is emphasized that malingering instructions were in effect during both the P300 tests, as well as during the TOMM sessions. This is detailed in the Supplementary Material.

### Data Acquisition

P300, measured P300 peak to the subsequent negative peak [“peak to peak” or p-p as in (27)] from Fz, Cz, and Pz, was recorded, filtered, artifacted, and averaged as previously [e.g., (28)]:

EEG recording used tin electrodes on the scalp at sites Fz, Cz, and Pz. They were referenced to linked mastoids. EOG was recorded with an electrode (tin) above the right eye and also referenced to the linked mastoids. Eyeblinks were removed with the method of Semlitsch et al. (29). Any remaining eye artifacts were manually detected, marked, and all trial data containing 80uV (or more) signals in any channel were dropped. The forehead was connected to the chassis of the isolated side of the amplifier (“ground”). Signals were passed through a Mitsar 19 channel (model 201) amplifier with a.16 Hz high pass filter setting, and low pass filters at 30 Hz. Output was conveyed to a 16-bit Mitsar Analog to Digital converter sampling at 500 Hz. For analyses and displays, single sweeps and averages were digitally filtered; the filter passed frequencies from 0 to 6 Hz using a *Kaiser* filtering algorithm. A minimum of 20 sweeps per average were required for each stimulus. The average number collected across subjects was 27.6 per subject.

P300 amplitude was measured at Pz using both the “base-to-peak” (b-p) and the (p-p) methods. [The p-p method has often been confirmed as the most accurate in P300-based deception studies: See (27, 30). Both b-p and p-p methods search from 300 to 650 ms for the largest positive 100 ms segment; this is the b-p P300. The midpoint of this segment is defined as the P300 latency. The average amplitude difference of the segment from the pre-stimulus baseline is defined as the base-peak value. For p-p, the algorithm likewise searches for the largest *negative* 100 ms segment between P300 latency and 1,300 ms and then subtracts the average amplitude of that segment from that of the maximally positive segment. Our present choice of a search window was made based on the grand average of all subjects in all conditions, the procedure recommended by Keil et al. (31).

### Within Individual Analysis: Bootstrapped Amplitude Difference Method

To determine if the P300 elicited by one stimulus is greater than that elicited by another *within an individual*, the bootstrap method (32) was used on the recording from Pz. The bootstrap method answers the question of whether or not the probability is more than 90 in 100 that the real difference between the average probe P300 and the average irrelevant P300 is > 0. However, for each subject, one has only one average probe P300 and one average irrelevant P300 available. Answering the question requires distinct distributions of *average* probe and *average* irrelevant P300s, and these distributions are unavailable. We thus bootstrap these distributions with the following procedure: An algorithm goes through the combined (probe-followed-by target in the CTP and probe-followed-by non-target in CTP) set (all single sweeps) and randomly draws, *with replacement*, a set of n1 probe waveforms. It averages these and computes P300 amplitude from this average using the segment selection method described for the p-p index. Next a set of n2 waveforms is drawn on a random basis *with replacement* from the set of irrelevant waves, from which an average P300 amplitude is calculated. The numbers n1 and n2 are the actual numbers of accepted probe and irrelevant sweeps for a given participant, but n2 is multiplied by a fraction (about.142 in the present report) which randomly reduces the number of irrelevant trials to within one trial of the n1. The computed irrelevant mean P300 is then subtracted from the comparable probe value, resulting in a difference value for a distribution that will contain 100 values after 100 iterations of the process just described. (*BSITERS* is the number of iterations in which probe P300 > Irrelevant P300; it must be 90 or more in this report for a knowledgeable decision). Multiple iterations yield differing probe-minus-irrelevant differences because of the sampling-with-replacement process. (We also use the mean of this 100-iteration difference distribution here as a dependent variable, *BSMEAN*).

### Dependent Variables

In evaluating group effects of the critical independent variables, two different and related dependent variables were utilized here. First is the Pz p-p P300 amplitude difference from our sample in microvolts between probe and irrelevant P300 averages, that is usually large in knowledgeable, but not unknowledgeable subjects. We also use BSITERS and BSMEAN, defined above.

### Group Statistical Analyses

ANOVAs and *t*-tests were used for group analyses. Effect sizes for *p* < 0.2 are reported using partial eta squares (*petasq*). These values can be benchmarked against Cohen's (33), pp. 278–280) criteria of small (0.01), medium (0.06), and large (0.14) effects, as reviewed by Richardson (34). Cohen's d is used for all *t*-tests. Guidelines for d are as follows: small (0.2), medium (0.5), and large (0.8). For 2-level independent variables in all cases of marginally significant effects (*p* < 0.15), Bayes factors [JZS BFs, with scaled r = 0.707, as in Rouder et al. (35); as obtained from http://pcl.missouri.edu/bayesfactor] are also reported here as “BFs.” The BFs are mostly used to confirm the likelihood of the null hypothesis (relative to the likelihood of the alternative hypothesis) when *p* > 0.05. This cannot result from non-Bayesian analysis. We do not use them when *p* > 0.25 or <0.01. BFs are mainly used to provide relative support for the null hypothesis, however when *p* > 0.25, the probability that an observed difference is due to chance is difficult to rule out so it becomes pointless to give the BF. Likewise, if *p* < 0.01, it is increasingly gratuitous to use BFs to help confirm the alternative. These BF likelihood ratios are stated as favoring the null or the alternative hypothesis, and the associated numbers will be odds ratios favoring either hypothesis. When these ratios are close to 1.0, they cannot be interpreted as favoring either hypothesis, as one is about as likely as the other.

Despite the often cited 57-year old interpretation (36) of the BF, this factor is a *continuous measure* and “does not force an all-or-none decision, but instead reallocates belief [in null vs. alternative hypothesis] on a continuous scale.” [from Schönbrodt et al. (37), p. 2]. One can never prove the Null Hypothesis, but the *continuous measure* perspective of the BF discourages arbitrary thresholds of confidence, although these are still often used. A recent treatment was provided by Kass and Raferty (38) who suggested that BF = 1–3.2 is worth a bare mention, BF = 3.2–10 is “substantial,” BF = 10–100 is “strong” and BF > 100 is “decisive.” In givinging a BF, we always divide null likelihood ratio by alternative if BF favors null, and we always divide alternative by null if BF favors alternative. Therefore, all our BFs are positive and equal to or >+1. When we state “The BF in this test was 2.5 in favor of the null” we mean that the null hypothesis is 2.5 times as likely as the alternative hypothesis. Likewise, “The BF in this test was 5.5 in favor of the alternative” means that the alternative is 5.5 times as likely as the null hypothesis. For higher (>2) level ANOVAs, in which the usual ANOVA yields an effect of interest with *p* < 0.2, we do Bayesian ANOVAs in JASP (https://jasp-stats.org/) in order to estimate evidence for the null relative to the alternative hypothesis.

#### Behavioral Results: TOMM Data

All behavioral and ERP data collected are in a SYSTAT 8.0 data file and may be obtained by contacting the senior author, jp-rosenfeld@northwestern.edu

We used the TOMM to establish (1) that malingering groups (simple, SM; motivated-paid, Bt $; and motivated-unpaid, BtNo) were malingering, as instructed, and (2) to establish that there were behavioral differences among groups attributable to the differing instructional sets heard by each group.

There is no question that all three groups were malingering. Using the Hilsabeck et al. (26) norms (>82/100 correct is normal/not malingering; 62/100 or less suggests malingering), all groups were malingering since no P scored more than 59 of 100 opportunities for correct responses.

**Figure 2:**
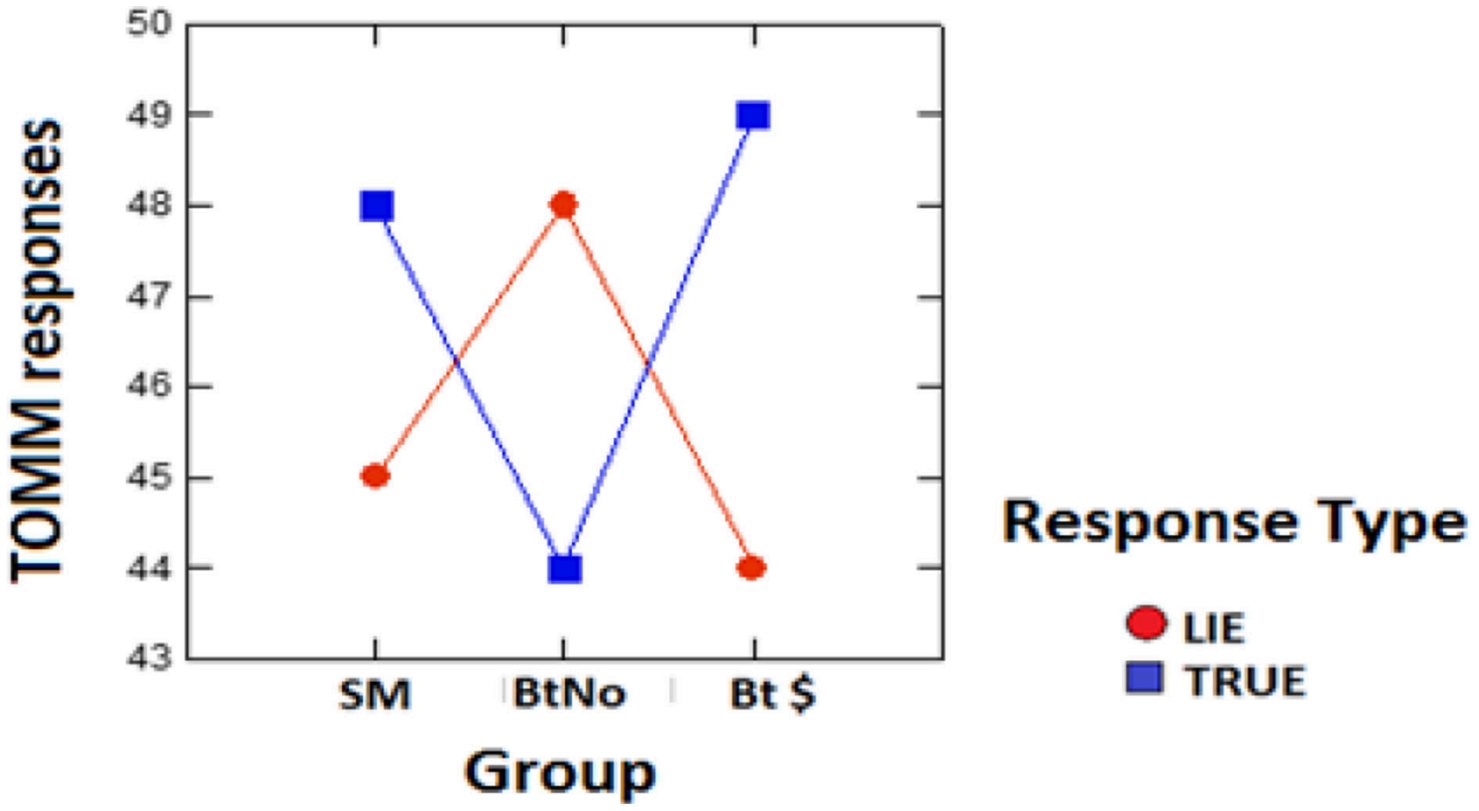
TOMM data. Numbers of correct/honest (“TRUE” in blue) and incorrect/malingered (“LIE” in red) responses in 100 trials as a function of group.

The number of correct/honest (“TRUE” in Figure 2) and incorrect/malingered (“LIE” in Figure 2) responses out of 100 total trials is shown as a function of incentive group in Figure 2. This figure and the subsequent statistical analyses are based on the full initial P number = 64, less four outlier subjects (one each from Groups SM and BtNo, and two from Bt $) whose correct response numbers were more than 2SD from the respective group mean.

Figure 2 shows what appears to be a complex interaction of group and response type. A 2-way, mixed 3 (groups) × 2 (response types) ANOVA was performed. The main effect of groups was ns at *F*_(2,56)_ = 0.091, *p* = 0.914. Likewise the main effect of response type was ns at *F*_(1,56)_ = 1.363, *p* = 0.248. These null effects were expected in view of the apparent interaction of group by response type. This effect was significant at *F*_(2,56)_ = 3.17, *p* < 0.05, petasq = 0.098 (medium to large). To follow up on this result, we decided to do one 2 × 2 ANOVA on a *post-hoc* basis, in which we compared only the paid vs. unpaid, both encouraged to beat the test without instructions as to how to do so. This would allow comparison with the same test done on instructed participants (also paid vs. unpaid) in Rosenfeld et al. (1). The 2 (groups, BtNo vs. Bt $) by 2 (response types, TRUE vs. LIE) ANOVA revealed no main group effect with *F*_(1,37)_ = 0.118, *p* = 0.733. Neither was there a main effect difference between numbers of TRUE vs. LIE responses; *F*_(1,37)_ = 0.13, *p* = 0.721. This also related to the significant interaction, *F*_(1,37)_ = 5.805), *p* = 0.021, BF = 2.84 in favor of the alternative (meaning that the alternative hypothesis of the interaction is 2.84 times as likely as a null effect), with petasq near large at 0.136. This significant interaction for the motivated *uninstructed* groups run here was exactly the same as the one reported in Rosenfeld et al. (1) for motivated *instructed* groups which were otherwise exactly like the *uninstructed* BtNo and Bt $ groups run here. We suggested then that the interaction is consistent with the view that the paid malingered group pays more attention to malingering instructions, by being more careful about not malingering too much. However, the present results suggest that this interaction does not depend on detailed malingering instructions, given that the specific malingering instructions were not used here, yet the same interaction was obtained.

The 2 × 2 interaction was decomposed by doing *t*-tests comparing TRUE and LIE responses within each group: Within the BtNo group, *t*_(18)_ = 1.231, *p* = 0.234, BF was null at 2.183. However, within the Bt $ group, *t*_(19)_ = 2.427, *p* = 0.025, BF supported alternative at 2.39. Thus, in the Bt $ group, the financial incentive was sufficient to produce the significantly greater number of truthful responses.

The results emphasizing that paid malingerers perform more accurately/honestly –*as instructed*–than unpaid malingerers is as we predicted, and as was seen in earlier studies of malingering reviewed in the introduction. The interactions and related results also confirm our manipulation regarding malingering.

By combining TOMM data from the *instructed*, motivated groups in Rosenfeld et al. (1) with the present TOMM data from *uninstructed* motivated groups, we found no effects in an ANOVA (2 groups × 2 response types, TRUE, and LIE) on combined instructed and uninstructed groups: For groups, *F*_(1,80)_ = 1.132, *p* = 0.29, BF favors null at 2.07 (i.e., the null is more than twice as likely as the alternative). For response types, *F*_(1,80)_ = 0.918, *p* = 0.341, BF favors null at 2.25 (i.e., the null is more than twice as likely as the alternative). Neither was the interaction significant; *F*_(1,80)_ = 0.174, *p* = 0.678, BF favors null at 3.02, which approximates the Kass and Raferty (38) criterion of “substantial” evidence for the null hypothesis. Thus, we saw no evidence supporting the effect of instruction on honest vs. dishonest behavioral responding.

#### Behavioral Results: Reaction Time Data

RTs to Probe and Irrelevant items in birthday and experimenter name conditions of the P300 CIT are shown in Table 1 for all three groups. We had no specific predictions about the effect of motivational manipulation on RTs, other than what might be predicted from Seymour et al. (39), i.e., that probe RTs would be longer than irrelevant RTs. Moreover, we found no group differences in the instructed groups of Rosenfeld et al. (1). Thus, we performed a 2 (stimulus types; probe vs. irrelevant) × 2 (memory types; name vs. birthdate) × 3 (group; SM vs. BtNo vs. Bt $) ANOVA. The effect of group was ns; *F*_(2,46)_ = 0.598, *p* = 0.554. The effect of memory type was likewise ns; *F*_(1,46)_ = 0.619, *p* = 0.435. The interaction of group and memory type was likewise ns, *F*_(2,46)_ = 0.332, *p* = 0.719. The main effect of stimulus type was also ns; *F*_(1,46)_ = 1.164, *p* = 0.286, nor did stimulus type interact with group; *F*_(2,46)_ = 0.158, *p* = 0.855. However, the interaction of memory type and stimulus type was significant; *F*_(1,46)_ was 10.294, *p* = 0.002, with petasq = large value of 183. The triple interaction was ns; *F*_(2,46)_ = 0.733, *p* = 0.486.

**Table 1:** Behavioral reaction times (msec) to probe and irrelevant birthdates (BD) and Experimenter Names (NM) during CTP.

**GROUP**	**PROBE BD**	**IRREL BD**	**PROBE NM**	**IRREL NM**
Unpaid	345.8	328.0	332.7	332.6
Paid	372.1	357.7	364.2	370.7
Simple malinger	388.7	377.7	362.8	363.5

We thus, re-examined effects within memory type by first performing a 2 (stimulus types) × 3 (groups) ANOVA on birthdate data only. The results were no group effect; *F*_(2,52)_ = 0.743, *p* = 0.481. However, we did find the predictable effect of stimulus type, with *F*_(1,52)_ = 8.57, *p* = 0.005, petasq = 0.141 (large), with BF substantially favoring alternative at 6.69. The interaction was ns at *F*_(2,52)_ = 0.161, *p* = 0.851. The same analysis on the name data yielded no significant effects: For groups, *F*_(2,47)_ = 0.402, *p* = 0.671. For stimulus type, *F*_(1,47)_ = 0.354, *p* = 0.555, and the interaction was *F*_(2,47)_ = 0.273, *p* = 0.763.

There are thus, in agreement with others [e.g., (11)], no effects of motivational group on RT; the familiar effect of stimulus type on RT (39) holds up, but only in the birthday data.

### Qualitative ERP Results

**Figure 3:**
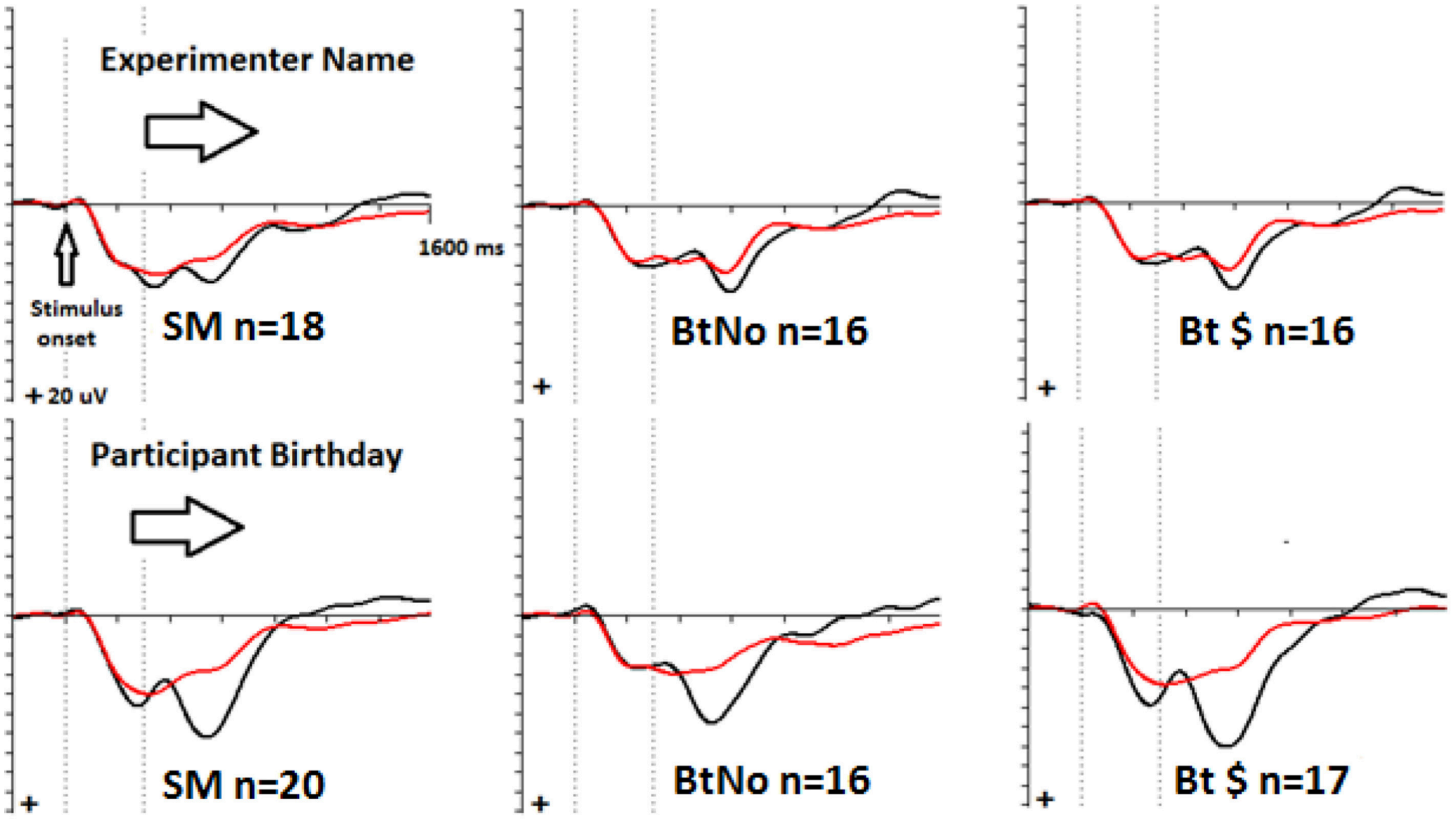
Averaged P300 response waveforms to probes (black font) superimposed on irrelevant (red font) P300s in 3 groups in 3 columns from left to right: SM, BtNo, and Bt $. The top row shows ERPs elicited by episodic experimenter name stimuli; the bottom row shows ERPs elicited by semantic participant name stimuli. The dashed vertical lines show stimulus onset and offset in temporal order.

**Figure 4:**
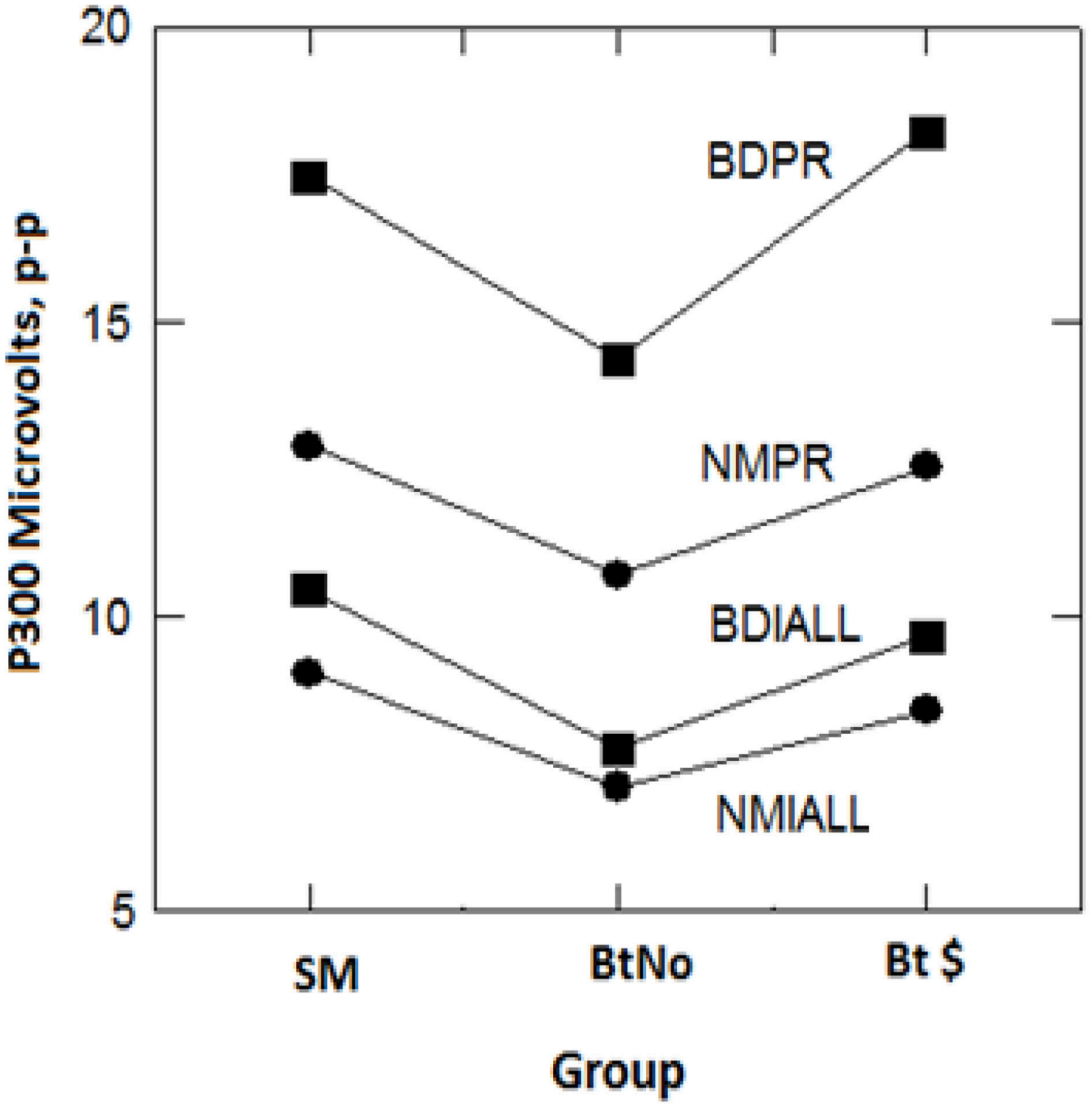
Computed P300 (p-p) values in microvolts as a function of groups on the x-axis for 4 stimulus/memory types: NM is experimenter name, BD is participant birthdate, PR is average probe, IALL is average of all irrelevants.

The grand average ERPs are seen in Figure 3, sorted by incentive groups (columns; Simple Malingering, “SM”, beat test without pay, “BtNo” and beat test for pay, “Bt $”) and memory types (rows; Top: experimenter's name/episodic vs. Bottom: participant's birthday/semantic). The visually obvious effects are probe P300 > Irrelevant P300, and birthdate probe-minus-irrelevant P300 > name probe-minus-irrelevant P300. Figure 4 shows a plot of computed P300 amplitude (p-p) as a function of group: (SM, BtNo, and Bt $), stimulus type (PR: probe vs. IALL: irrelevant), and memory type (name, NM vs. birthdate, BD). (IALL is the average P300 of all irrelevant P300s).

### Quantitative ERP Results

Of the 64 initially run participants in three groups, the analyses and figures below are based on 46–53 participants (depending upon whether name or birthdate stimuli were involved. Members of the SM, unpaid (BtNo) group and of the paid (Bt $) group had either birthdate and/or experimenter name data removed due to excessive artifacts, or in one case, failing to follow instructions. Thus, between-group analyses were based on at least 18 SM, 13 unpaid, and 15 paid subjects, cell sizes we have used in multiple previous studies [determined via a priori power analysis, and as reviewed in (40)].

Following up on Figure 4, we first did a 3-way, 2 (stimulus types, probe vs. irrelevant) by 2 (memory type, episodic vs. semantic) by 3 (groups, SM, BtNo, Bt $) ANOVA; the “Bt” notation means both groups were motivated to *beat* the *test*. As we found in Rosenfeld et al. (1) with a different group of instructed participants, there was the usual main effect of stimulus type with *F*_(1,44)_ = 145.1, *p* < 0.001, petasq = 0.767, and a main effect of memory type, *F*_(1,44)_ = 22.1, *p* < 0.001, petasq = 0.34. The interaction of stimulus type and group was ns with *F*_(2,44)_ = 0.47, *p* = 0.628, petasq = 0.02. The interaction of memory type and group was ns with *F*_(2,44)_ = 1.19, *p* = 0.313, petasq = 0.05. As in Rosenfeld et al. (1), we also saw a significant interaction of stimulus type and memory type, *F*_(1,44)_ = 22.6, *p* < 0.001, petasq = 0.34, indicating a greater effect of stimulus type for semantic than for episodic memory type. This 2-way interaction, evident from Figure 4, shows that the probe-irrelevant differences were greater for the birthday (semantic) than experimenter name (episodic) stimuli across all three groups, SM, BtNo, and Bt $. This was confirmed in a follow-up ANOVA in which the dependent variable was probe-irrelevant P300 difference as a function of memory type and group. The effect of group was again ns, *F*_(2,53)_ = 0.799, *p* = 0.455. The critical effect of semantic vs. episodic memory type was *F*_(1,53)_ = 48.94, *p* < 0.001 with petasq = 0.46, a very large effect. The interaction of memory type and group was just short of significance, *F*_(2,53)_ = 2.795, *p* = 0.07.

The triple interaction was clearly not significant, *F*_(2,44)_ = 0.289, *p* = 0.75, petasq = 0.01. The main effect of group was marginally short of significance with *F*_(2,44)_ = 2.85, *p* = 0.069, BF = 1.34 (indeterminate), petasq = 0.115, probably reflecting the fact that the BtNo group showed slightly reduced P300s across all stimuli in Figure 4 for unknown reasons. However, this non-significant effect is of minor interest in this study; our main interest concerns effects of motivational group on the *CIT effect*, i.e., the probe-irrelevant P300 (p-p) amplitude difference, and that is reflected by the non-significant interaction of stimulus type and group, described above as *p* = 0.628. This was not the case for the behavioral/TOMM data in which Figure 2 and its analysis showed a clear difference between paid and unpaid groups: The interaction term in that analysis meant that the difference between probe-irrelevant differences was significant at *p* = 0.02, with a BF of 2.84 in favor of the alternative. To compare P300 data, we did a *post-hoc* comparison (*t*-test) restricted to paid vs. unpaid groups' probe-irrelevant P300 differences (name and birthday combined) from Figure 4. The result was *t*_(36)_ = 0.438, *p* = 0.664, BF favoring null at 2.67.

In view of the significant effect of memory type, we decided to do follow-up, separate analyses within memory type, and the dependent variable we used was in all cases the CIT effect, i.e., the probe- irrelevant p-p P300 difference: In these follow-up tests, we planned *a priori*, orthogonal comparisons, namely, (1) the comparison of the SM group with both combined motivated groups (paid and unpaid), and (2) the comparison of paid and unpaid groups. For the episodic experimenter name stimuli, the comparison of SM with both motivated groups combined was ns, *t*_(47)_ = 0.038, *p* = 0.968, d = 0.012, BF = 3.4, substantial evidence (38) in favor of null. For comparison of the two motivated groups, likewise, *t*_(29)_ = 0.386, *p* = 0.703, *d* = 0.139, BF = 2.77, which is close to substantial evidence in favor of null, and is close to the null hypothesis being three times as likely as the alternative hypothesis. For the semantic birthdate stimuli, the comparison of SM with both motivated groups combined was ns, *t*_(52)_ = 0.462, *p* = 0.646, *d* = 0.126, BF = 3.25, which is substantially in favor of null. For comparison of both motivated groups, *t*_(32)_ = 1.623, *p* = 0.114, *d* = 0.557, BF = 1.12 in favor of null, although this low value provides clear support for neither null nor alternative hypothesis. Over all these comparisons, there is scant support for the effects of financial motivation and incentive to defeat the test on the P300-based CIT effect.

**Figure 5:**
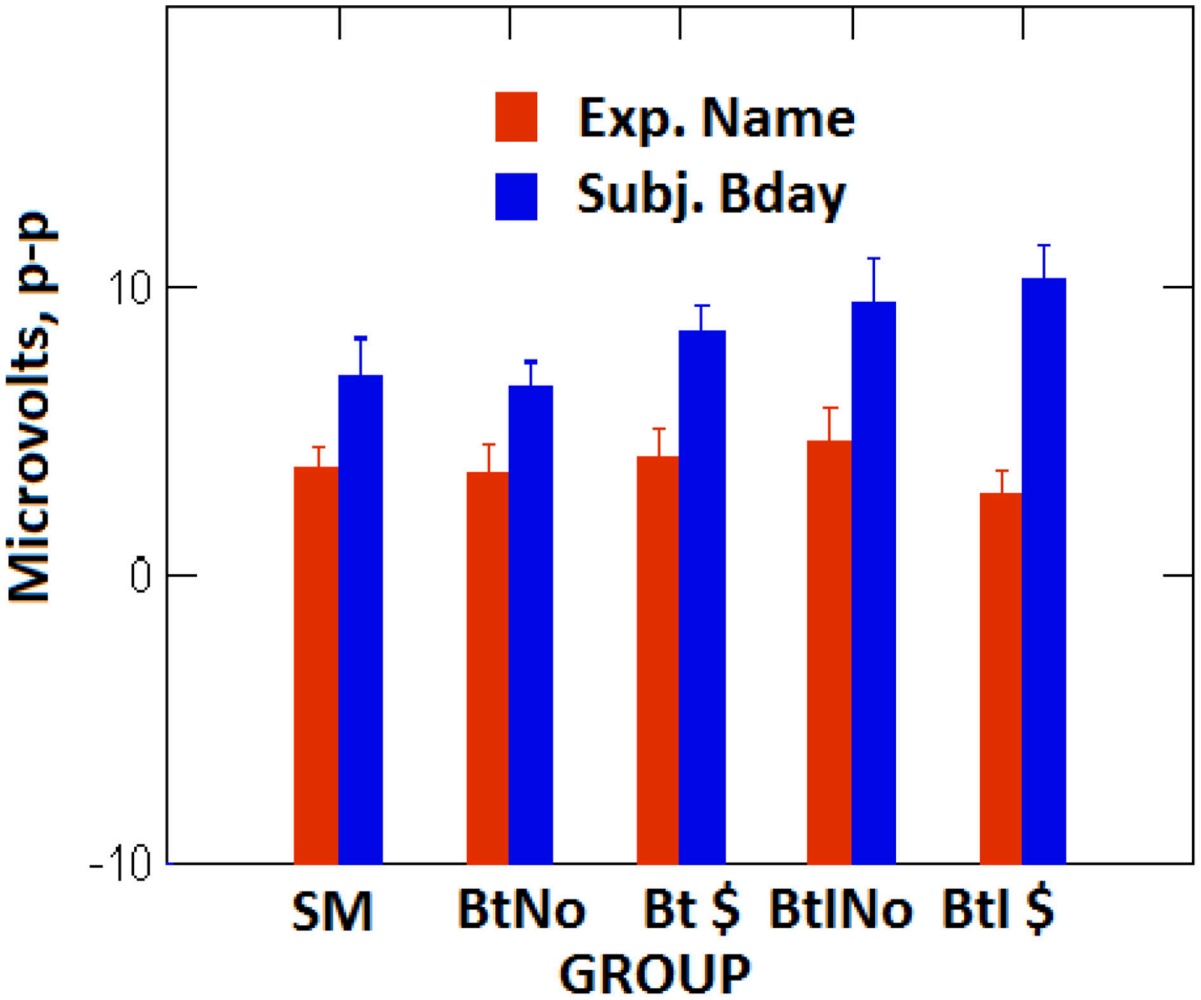
Computed probe-minus-irrelevant P300 (p-p) difference values in microvolts as a function of 3 groups on the x-axis, as in Figure 4, supplemented by 2 instructed groups (BtINo and BtI $) from Rosenfeld et al. (1). Experimenter name values are in blue, participant birthdate values are in red. Error bars are S.E.M. values.

In Rosenfeld et al. (1) there were also two motivated malingering groups, one paid and one unpaid, but both were additionally instructed how to beat the test (on the same stimuli as used here). It is thus possible to combine that data set with the present one, and thereby obtain the isolated effect of instructions. Figure 5 shows a bar graph of the five groups run in both the present and previous studies, the latter groups italicized in the following list: (1) the simple malingering (SM) group, (2) the uninstructed, unpaid group motivated to defeat the test (BtNo), (3) the uninstructed, paid group motivated to defeat the test (Bt $), (*4) the instructed, unpaid group motivated to defeat the test (BtINo), and (5) the instructed, paid group motivated to defeat the test (BtI $)*. To examine the effect of instructions, we compared the combined second and third groups (both uninstructed) with the combined fourth and fifth groups (both instructed). For the name stimuli, *t*_(68)_ = 0.042, *p* = 0.967, *d* = 0.01, BF = 4.04, substantial evidence in favor of null. However, for semantic birthdate stimuli, *t*_(72)_ = 2.07, *p* = 0.04, *d* = 0.505, BF = 1.48 anecdotally in favor of alternative. As Figure 5 suggests, for semantic birthday stimuli the probe-irrelevant difference for the two instructed groups at right is greater than for the comparable uninstructed groups, second and third from the left. So while we saw no effect of financial motivation on P300, we did see an effect of test-beating instruction.

Finally, although we showed separately within instructed (1) and uninstructed (above) groups, that financial motivation does not impact the CIT effect, we can now combine data from the previous and present studies (as in Figure 5) to do a more powerful test on the same issue. Thus, we compared the two combined motivated paid groups from Figure 5 with the combined motivated unpaid groups from the same figure. For the episodic name stimulus, *t*_(68)_ = 0.84, *p* = 0.404, *d* = 0.20, with BF substantially favoring null at 3.01. For the semantic birthday stimulus, *t*_(72)_ = 1.13, *p* = 0.263, *d* = 0.26, with BF favoring null at 2.4; i.e., null is 2.4 times as likely as alternative. This supports the *lack of effect of financial motivation on the CIT effect for episodic as well as semantic stimuli*.

**Figure 6:**
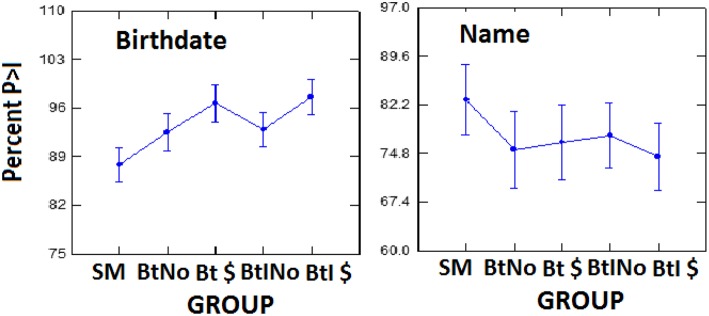
Percent of iterations in which probe P300 > irrelevant P300 as a function of group (as in Figure 5. and memory type; left box for birthdate stimuli, right box for name stimuli. The error bars for each panel show the mean SEM averaged across groups, so are all the same. There were actually differences among group SEMs.

Bootstrap-based individual diagnostic data are shown in Figure 6. The averaged, within-subject percentage of total iterations in 100 in which the probe P300 > Irrelevant P300 is shown on the y-axis, with incentive group, as in Figure 5, on the x-axis. Semantic birthdate-evoked values are at left, and episodic experimenter name-evoked values are at right. Consistent with the amplitude data described above, the hit rates are greater for semantic birthdate stimuli (at about 93% overall) than for episodic name stimuli (about 77% overall), nor does there seem to be much of a systematic main effect of group, with birthdate values slightly increasing across groups, while name values decrease. Although the y-axis ranges of both birthdate and name boxes are about the same (35–37, respectively), the error bars representing S.E.M. appear greater for name values than for birthdate values. Formal analysis of this effect is in Table 2, which shows variability indices in the five groups for the bootstrap iteration scores varying between 0 and 100%. Range refers to maximum score minus minimum across 100 iterations within Name (Nm) and Birthdate (Bd) conditions. The *F*-values are the variance ratios (distributed as F) of Bd divided by Nm. It is seen that Nm percentage variances are significantly smaller than Bd values in all four motivated groups, but not in the SM group. Likewise, there is no overlap between mean Nm and Bd range values in the motivated groups.

**Table 2:** Variability of bootstrap number (number of bootstrapped iterations in which P>I in 100 trials) across motivational groups from Rosenfeld et al. (1), BtINo and BtI $ (both instructed); and present uninstructed groups: SM, BtNo, and Bt $.

**Group**	**(*n*)**	**Nm range**	**BD range**	***F***	***p***
SM	(20)	53	61	1.14	ns
BtNo	(17)	23	79	9.45	<0.01
Bt $	(17)	20	77	22.3	<0.01
BtlNo	(20)	49	73	2.24	<0.05
BtI $	(20)	20	89	22.8	<0.01

*Range refers to maximum minus minimum in the group from 0 to 100 for name (Nm) and birthdate (BD) stimuli. F is the variance ratio from dividing BD variance by NM variance, with associated probabilities (p)*.

The first analysis of the data in Figure 6 involved a 2 (groups) by 2 (memory types) ANOVA. The two groups compared were the SM group vs. the four combined motivated groups. As predicted there was no main effect of group, *F*_(1,83)_ = 0.009, *p* = 0.927, petasq = 0.0001. Consistent with amplitude data and visual impressions, there was a main effect of memory type, *F*_(1,83)_ = 13.17, *p* < 0.001, petasq = 0.137. There was also a significant interaction, *F*_(1,83)_ = 4.87, *p* = 0.03, petasq = 0.05, confirming that the birthday percentages followed a different trend than the name values. We therefore next did separate *t*-tests within memory type, in which we compared SM with motivated groups as in the first ANOVA. For Birthdate (Figure 6, left), *t*_(23)_ = 1.937, *p* = 0.065, with the BF indeterminately favoring the alternative at 1.24 with Cohen's d = 0.55. For Name (Figure 6, right), *t*_(86)_ = 1.202, *p* = 0.233, with BF favoring Null at 2.051. Cohen's d = 0.358. There was thus no clear and consistent support for the notion that motivated groups perform differently than the simple malingering group. Finally, we analyzed for possible differences among the four motivated groups, separately within memory type. A 1 by 4 group ANOVA on the name data, with the dependent variable being number of P>I iterations in 100 yielded *F*_(3,66)_ = 0.064, *p* = 0.979, petasq = 0.003. For the birthdate data, *F*_(3,70)_ = 1.392, *p* = 0.253, petasq = 0.0563. On the bootstrapped iterations variable, within each memory type, there is no clear evidence of an effect of financial incentive.

## Discussion

A possible limitation on the conclusiveness and generality of the presently observed lack of support for motivational effects on the P300 CIT effect in the malingering scenario concerns the possible lack of statistical power available given the numbers of subjects utilized, i.e., 13, 15, or 18 per group. Although many of our previous ERP studies [see (7) for review] have utilized 12–15 subjects per cell, based on power planning analyses, and reported many robust effects, researchers used to group sizes of 20 or more may have reasonable concerns regarding some of the null ERP findings reported here. These concerns may be tempered, however, by the fact that the motivational manipulations which had negligible effects on P300 here, nevertheless had clear behavioral effects here in the same subjects. Moreover, we did make use of Bayes Factors, which allow one to quantify the relative likelihood of null and alternative statistical hypotheses. These values clearly favored the alternative hypotheses regarding the TOMM test effects, but favored the null hypotheses at near to and at substantial levels regarding P300 effects.

Another limitation on the generality of these results concerns the fact that the age range of participants was narrow (17–22). A future study can remedy this limitation by using the same methods with a sample of participants from a wider range of ages.

The present finding that financial incentive at levels that do produce behavioral effects, but do not appear to affect the P300 CIT effect in the CTP version of the P300-based CIT (for detection of malingering) is consistent with what we found previously (1, 12) using both the older 3-stimulus protocol, as well as the Complex Trial Protocol (CTP): (1) Both the older and present reports found this lack of financially motivated influence with both episodic and semantic memory stimuli, and (2) Semantic memory—evoked P300s are larger than Episodic memory-evoked P300s. In order to support the null findings on the incentive effect, which conflict with most findings on the SCR-based CIT (5), but are consistent with findings in the RT-based CIT (9, 11), it is essential (as emphasized by these authors regarding their manipulation check) to establish that the financial manipulation here produced credible behavioral effects.

We used the objective *test of memory malingering* [TOMM; (18)] to establish that: (1) both paid and unpaid groups malingered, and that (2) there were differences in malingering among groups. All three malingering groups here did indeed malinger, in that their correct response percentages were well-below the 82% cutoff for non-malingering behavior [and <62% indicates malingering; (26)]. Furthermore, the 3 × 2 ANOVA on Figure 2 showed that behavioral responses differed across groups as revealed in the interaction of response type and group. Moreover, the further *post-hoc* analysis of Figure 2 yielded a significant interaction that was exactly the same as that found in Rosenfeld et al. (1) with *instructed* subjects. This established that the financial incentive did create a behavioral effect in the present paid group that differed from the effect in the present unpaid group. Furthermore, since the same interaction obtained with or without detailed instructions [used in (1)] on how to beat the test, those instructions are apparently unnecessary for the interaction to obtain.

The instructions used in Rosenfeld et al. (1) emphasized that in order to malinger effectively, (i.e., to imitate the performance of a truly head-injured person), a participant would have to score about 50% correct and 50% incorrect responses. Thus, one would need to take care not to make too many errors. We suggested in Rosenfeld et al. (1) that a paid instructed subject would be more motivated to attend to the instructions than an unpaid subject, and thus not make too many errors, which would explain why they had more correct than incorrect responses in contrast to their unpaid counterparts. However, in the present study, the specific instructions (to approach 50% accuracy) were omitted, yet the present *uninstructed* participants closely approximated the performance of the previous *instructed* participants. The present participants were simply told “Although you are, of course, normal and have NOT suffered memory loss, your goal during all today's tests is to play the role of a head injured individual who has suffered traumatic brain injury. In other words you are to try to look and act as though you have suffered memory loss due to brain damage from an accident.” Apparently, more explicit instructions to approximate 50% accuracy rates were unnecessary to achieve rates near 50% accuracy, in that both the present paid and unpaid participants performed at near 50% accuracy (see Figure 2) i.e., between 44 and 49%, with the paid subjects showing a significant difference between correct/TRUE and incorrect/LIE responses (correct > incorrect), unlike their unpaid counterparts.

Indeed the lack of behavioral effect of specific 50% accuracy malingering instructions was further supported by the direct comparison of combined paid and unpaid instructed groups from Rosenfeld et al. (1) with the present paid and unpaid uninstructed groups: In that analysis, there were no effects in the TOMM scores, and all p's were >0.29 with all Bayes Factors supporting Null at values from 2.07 to 3.02. The lack of effect of specific 50% accuracy instructions on malingering performance was all the more interesting in view of the fact that with BD (although not NM) stimuli, the *instructions* do increase the P300 CIT effect with a medium effect size (*d* = 0.505). One explanation is that the instructions could have increased attention levels during the P300 CIT, which would lead to larger probe P300s (21). Thus, the TOMM seems to be a test of malingering, not attention, whereas, P300 is sensitive to attentional variables.

Why is it that SCR measures, but not RT-based nor P300-based CIT measures, are affected by financial incentive manipulations? As noted, the lacking effect of financial incentive could be attributed to not enough statistical power. Kleinberg and Verschuere (9) noted this possibility regarding their lacking effects of financial incentive on RT indices of the CIT effect. However, given that theirs was an internet study with many subjects, inadequate power seemed unlikely. ERP studies cannot be run at present on the internet, so we elected the *n*-values in the present study and in Rosenfeld et al. (1), based on power analysis. We supported our lack of effects with Bayes Factors (BFs) that allow statements about the likelihood ratios of null to alternative hypotheses. Given that these null effects of financial incentive on the P300 CIT effect are consistent with the results of Ellwanger et al. (12) using the 3-stimulus protocol, and of Rosenfeld et al. (1) using the complex trial protocol, we feel it reasonable to conclude that the financial incentives at levels utilized here do not appreciably influence P300-based indices of the malingering of cognitive deficit. However, effects of incentives of a magnitude used in field situations, cannot yet be ruled out. Again, these results, do not necessarily apply to the classical forensic CIT scenario.

Kleinberg and Verschuere (9) suggested that whereas, the ANS (SCR) CIT effect is more likely related to the Orienting Reflex (41), the RT CIT is instead more likely related to inhibitory processes and to response conflict. Likewise, the P300 CIT effect appears to be based on the simply cognitive phenomenon of recognizing rare, meaningful information (42). P300 amplitude is also associated with the amount of focused attention to stimuli (21). This suggests that since a financial incentive should increase attention [confirmed in the TOMM test here and in (1), with the finding of fewer error/deceptive trials in paid Ps], the incentive manipulation should also produce larger P300s to familiar stimuli. However, once attention is enough to assure recognition of probes within a memory type category, the resulting P300s consequently generated in a more all-or-none manner are no longer influenced by motivation. Apparently, in the present study as in Rosenfeld et al. (1), attention to stimuli was adequate to assure recognition, whose consequent P300s, were no longer modifiable by motivation.

Moreover, as noted above, paid Ps appeared more motivated to follow self-imposed instructions suggesting that the best way to convincingly appear head injured was to not miss *all* test items, but to try to balance honest and dishonest responses during the P300 test. However, if this was the case in the present paid Ps, they would be experiencing a greater workload during the CIT, tending typically to reduce P300 amplitudes and CIT responses—which we didn't observe here. There are thus many complexly organized psychological factors with many neural substrates interacting to yield the present effects, and it is clear that much more research will be required to fully account for the present lack of effects of financial incentive in the P300 CIT.

A critical remaining question is: Why do *uninstructed* malingerers behave as if they were instructed to approximate a 50% accuracy rate? Perhaps in the absence of specific instructions, the *default* response style is to not respond falsely on all trials. More likely, the present instructions could have inadvertently suggested or implied an accuracy rate closer to 50% than to 0%: Both BtNo and Bt $ groups were told, prior to the P300 CIT: “Your goal is to produce the disability in such a way that the examiner would not know you are faking or pretending.” Prior to the TOMM, these same subjects were told, “your goal is to produce the symptoms of the disability, so we ask you to keep pretending that you are suffering memory loss and thus not able to recognize some of the pictures, and therefore to not press all the response buttons correctly.” Such explicit instructions could easily have served to implicitly warn participants not to press all buttons *incorrectly* also. In contrast, as is next discussed, in the previous SCR-based forensic CITs, participants are directed to respond falsely to all probe-type trials.

We have noted here that the present head injury malingering scenario differs from the mock crime-forensic scenario. Perhaps this difference is the reason why financial incentive affects SCR-based forensic scenarios but not P300-based malingering scenarios. The previous SCR studies typically gave test-beating instructions emphasizing that Ps not respond to *any* crime-relevant probe stimuli, e.g., “You are about to take a polygraph test in which enhanced responses to the critical item would indicate guilt. Your task is to avoid being detected and if you beat the test and are classified as innocent, you will receive a cash reward of $10” [This was based on a review of the original submission of (1), by Gershon Ben Shakhar]. In contrast, as noted above, the incentivized participant in Rosenfeld et al. (1) was instructed to try to duplicate the behavior of actual head injured patients, who do *not* fail to respond to all critical probes, but to only about 50% of them. This is the typical strategy of instructed simulated malingerers in most of the numerous head injury malingering studies [see (43)], including our Ellwanger et al. (12) study, although as noted already, the specific malingering instructions were omitted in the present report. Nevertheless, the present participants behaved as if they were following such an instruction set, perhaps self-imposed. It therefore is not clear that results of this malingering strategy (of not making 100% errors) are strictly comparable to those strategies used earlier (“don't respond to *any* probes”) to defeat a classical SCR-based CIT of the older ANS studies based on a mock theft scenario. Nevertheless, it is certainly clear from the present dataset and from Rosenfeld et al. (1) that financial incentive does not influence P300 in malingering performers. Moreover, we have now run a classical *mock theft scenario* using the CTP with participants motivated to beat the test, with one group paid and the other unpaid to beat the test (as in the present malingering study), and reported that (44) there was no effect of financial incentive on the P300 CIT in mock crime performance, just as with the present malingerers. Increasingly, the lack of effect of financial motivation on the P300 CIT effect becomes clearer.

As has been long argued [e.g., (45)], semantic information is stored more powerfully than incidentally acquired episodic information. The present results, along with the previous Rosenfeld et al. (1) results, are quite consistent with that notion. First, the probe-irrelevant differences and probe P300s are clearly larger with participant birthday stimuli (BD) than with experimenter name stimuli (NM). However, probe-irrelevant P300 differences with NM stimuli, however reduced, were seen here in contrast to RT effects, suggesting a greater sensitivity of P300 to weak memory traces, than of RT. Second, we did observe a significant effect of malingering instructions on BD-evoked but not NM-evoked P300s and RTs. Third, our bootstrap data showed expectedly higher detection rates for BD data than for NM data. Moreover, the effect of motivational and instructional incentives on BD detection rates were clearly different than on NM detection rates (Figure 6). This may be related to the greater variability seen for NM than BD bootstrap scores (Table 2), although, remarkably, not seen in the P300 data. It appears that participants are more uniformly detected with semantic than with episodic stimuli: Participants' detection scores cluster in a narrow range above 90% detection with semantic stimuli, but vary across a wide range with episodic stimuli. This implies that semantic stimuli are recognized on many more trials than are episodic stimuli.

It may seem surprising that financial incentive has no *incremental* effect after participants are instructed to defeat the test. This may be since our reward of $10 (US) for beating the test may be too inadequate to appeal to our mostly upper class undergraduates at a prominent private university. On the other hand, perhaps the intellectual challenge suggested by inviting participants to defeat the test may be more motivating than financial reward. This is an empirical question. Nevertheless, it is not unreasonable to conclude that the effect of financial reward is less in the P300-CIT [both forensic and malingering scenarios; (44, 46)] than in the autonomic CIT, since in the latter, similarly small rewards do in fact affect detection when added to instructions to beat the test (5, 47). This is important because it suggests that findings with student participants in university settings may well be applicable to field situations with higher stakes.

## Data Availability

All datasets generated for this study are included in the manuscript and the Supplementary Files.

## Ethics Statement

This study was carried out in accordance with the recommendations of Northwestern University Institutional Review Board with written informed consent from all subjects. All subjects gave written informed consent in accordance with the Declaration of Helsinki. The protocol was approved by the Northwestern University Institutional Review Board.

## Author Contributions

All authors listed have made a substantial, direct and intellectual contribution to the work, and approved it for publication.

### Conflict of Interest Statement

The authors declare that the research was conducted in the absence of any commercial or financial relationships that could be construed as a potential conflict of interest.
