# Supplementary material for: Financial Incentive Does Not Affect P300 in the Complex Trial Protocol (CTP) Version of the Concealed Information Test (CIT) in Malingering Detection. II. Uninstructed Subjects
Source: Front Psychiatry. 2019 Apr 15;10:189. doi: 10.3389/fpsyt.2019.00189 (PMC6476237; doi:10.3389/fpsyt.2019.00189)
Supplement: Supplementary file 1 [file Data_Sheet_1.docx]

**SUPPLEMENTARY MATERIALS:**

1. **Instructions to all Ps:**

**“Hi, come on in. My name is Elena. I emailed you about our appointment. I am going to hand you a set of instructions for what we will do today.”**

**“First, thank you for participating in our research. Now you need to read and sign this consent form.”**

***(Give consent form to subject).***

**“Any questions?”**

***Answer questions.***

***Consent form signed.***

**"Now I will play the information about the project we are researching, and you will read it at the same time."**

***Give written instructions to subject & play recording made by senior author.***

**[Recording ]**

**“Let me tell you something about the project we are researching. We are studying to see if it is possible to successfully pretend you have a memory problem following an accident involving a head injury. This is a real problem in the clinical field of Neuropsychology. Mild and moderate head injuries usually do NOT produce memory and thinking problems, but this rarely does happen. Much more common is the case of malingering. That is, some people who have had a mild or moderate accident involving the head, falsely and dishonestly claim all kinds of cognitive (memory) problems and sue insurance companies for large money settlements to compensate them for claimed lost ability to work, and so on. We want to compare the memory performance of both honest individuals as well as fakers on our special memory tests.” *For all subjects we add:* “In the next time period you will be “a faker”. You will be given brain wave tests of memory, as well as a special memory test.”**

**“Any questions?”**

***Answer questions.***

***Give to subject the form with dates and names and the copy of the CIT instructions.***

1. **FORM**

**Subject’s Name _________________________________________ File Name ______________________**

**Last First**

**What is your Birthday? __________________________________________________________**

**Month/Day/Year**

1. **Remove familiar irrelevants**

**Please mark any of the dates or names below that are familiar or relevant for you. “Relevant” meaning your mom’s birthdate or name or friend’s or other relatives’**

**1. Aug 4 1. Anne**

**2. Feb 9 2. Carol**

**3. May 30 3. Susan**

**4. Oct 12 4. Betty**

**5. Jun 27 5. Nancy**

**6. Sep 23 6. Mary**

**7. Jan 8 7. Liz**

**8. Apr 11 8. Alice**

**9. Nov 6 9. Margaret**

***Subject fills out the form.***

**“Thank you!”**

**"Now I will tell you the detailed instructions as you read them".**

1. **Instructions:**

**All the following (based on Ellwanger et. al., 1996 and Rosenfeld et al., 2017) is communicated to all Ps, with the exception that underlined text is given only to motivated, unpaid (BtNo; told to try to beat the test) subjects, and *italicized* text is given to paid, motivated subjects (Bt $; told to beat the test and paid for doing so). Text which is both italicized and underlined is given to both BtNo and Bt $ (but not SM) groups:**

**“You are about to undergo some memory tests. These tests will be administered on the computer and will be monitored by an examiner. These tests are extremely important to our understanding of impaired memory. Some individuals who have accidents are normal, or unharmed, following their accidents, but nevertheless may fake injury to obtain financial awards. We want to know what this type of faked memory performance looks like.**

***Although you are, of course, normal and have not suffered memory loss, your goal during all today’s tests is to play the role of a head injured individual who has suffered traumatic brain injury. In other words you are to try to look and act as though you have suffered memory loss due to brain damage from an accident***

***Your goal is to produce the disability in such a way that the examiner would not know you are faking or pretending. If you were caught faking a memory disorder in real life it could be considered a crime and you could be prosecuted.***

**In this experiment, electrodes will be placed on your head and earlobes. A harmless conductive paste is used to apply the electrodes; it can be easily cleaned off. A sink, water, and towels are available in the lab if you wish to clean yourself immediately afterwards.**

**Do NOT wear contacts. If you need glasses, you MUST be sure to wear them, not contacts.**

**You will be seated in a comfortable chair while a series of stimuli are presented on a screen in front of you. The stimuli will be presented in pairs. Each pair includes a date (or a name) – that would be the first stimulus in a pair and it will be followed by a string of numbers 11111, or, 22222, or, 33333, or, 44444, or, 55555) – that would be second stimulus in the same pair. There will be 210 pairs presented in a run.**

**THE FIRST STIMULUS. When you see first stimulus in a pair, you have to immediately press the RIGHT button using your index finger on the LEFT-HAND mouse to indicate that you have seen the name (or date). Some dates and names might be familiar or relevant to you, but others will have no relevance to you. No matter if a date or name is relevant or irrelevant to you, you should react to all of them exactly the same way (with a right button press on the left mouse).**

**Remember, no matter which stimulus is presented, you always want to press the button as soon as possible.**

**THE SECOND STIMULUS, which you see a bit later in the trial, will be a string of numbers and it will appear soon after the first stimulus. The 11111 string is your target. When you see the target string 11111, respond immediately by pressing the RIGHT (“YES”) button on the RIGHT-HAND mouse. If the second stimulus is 22222, or 33333, or 44444, or 55555, you press the LEFT (“NO”) button on the RIGHT-HAND mouse.**

***Once the task begins, please pretend and perform as if you have a significant memory disorder while taking all of the memory tests.* *Imagine that if you pretend well enough, you will be given a large sum of money by an insurance company because of this “injury”. In actuality you can earn $10 if you successfully “beat” all our tests to the experimenters’ satisfaction, as the result of inspection of your brain wave record at the end of the experiment.***

**PAY ATTENTION TO ALL STIMULI! At a few random times during testing, the experimenter will pause the stimulus presentation and ask you to identify the last stimulus you saw on the screen. More than one incorrect identification will result in unusable data and early termination of your participation, with no possibility of a reward.**

**It is crucial that you control your blinking, trying to reduce it as much as you can. If needed, you can blink when you see numbers (not when your see dates or names).**

**Remember: during the course of a trial, keep your eyes focused on the center of the screen where the stimuli appear. Please try to sit still, relax all your facial and body muscles and try to minimize blinking.**

**Thank you!”**

**“Any questions?”**

**_______________________________________________________**

**The subject then repeats back what he/she is supposed to do as a subject in this experiment.**

**P is asked before NAME bloc, “Do you remember my name? What is it?”**

**If P says “YES, Elena”, we continue with the experiment. If “NO”, Experimenter shows name again on a card.**

**Experimenter puts cap on the subject, fills electrodes with gel, and attaches reference electrodes to mastoids. Also an electrode would be placed supra-orbitally (FP1) for EOG recording.**

**Turn off light.**

**Start the experiment.**

**We alternate (subject to subject) dates and names presentation for first ERP block.**

1. **TOMM session*:***

**TOMM instructions given to all subjects and we ask him/her to read along as experimenter speaks.**

**STUDY PHASE**

**In this part of the experiment you will see 50 pictures, one at a time. Each pictures appears on the screen for just 3 seconds. Look at each one and try to remember it.**

**TEST PHASE**

**“Now you are going to see more pictures and respond to each of them with a button press.**

***Remember, your goal during these tests is to play the role of a head injured individual who has suffered traumatic brain injury.***

***You are to try to look and act as though you have suffered memory loss due to brain damage from an accident.***

**If you were acting honestly, your task would be:**

**1) . A button immediately, if you recognize a picture on the screen.**

**2) To press B button if you don’t recognize a picture on the screen.**

***But, your goal is to produce the symptoms of the disability, so we ask you to keep pretending that you are suffering memory loss and thus not able to recognize some of the pictures, and therefore to not press all the response buttons correctly*”.**

**“Any questions?”**

**Answer questions.**

**If the subjects are clear and don’t have questions, the TOMM is given.**

**When the TOMM is over, we inspect the erp data and decide if a Bt $ P should be paid. We say to the subject:**

**”*Now we will generate, then look at-- your brain waves on the computer display to see if you beat the brain wave tests both on birthdays and names. I will tell you that if you beat the test, one brain wave in solid black will look similar to the second brain wave in dotted line. But if there is a big difference, then we caught you and you do not get the reward. See below for example.”***

***Experimenter now points to a picture to show a miss and a catch.***

**________________________________________________________**
